# Comparative study of noninvasive chromosomal screening using blastocyst culture media: frozen–thawed embryos outperform fresh embryos

**DOI:** 10.3389/fendo.2026.1851046

**Published:** 2026-06-16

**Authors:** Xiaojun Wen, Junye Huo, Zhanhui Ou, Xiufeng Lin, Wanna Ke, Yanjun Lyu, Qingni Li, Xubin Zhang, Zhiming Li

**Affiliations:** Reproductive Medicine Center, Boai Hospital of Zhongshan, Zhongshan, Guangdong, China

**Keywords:** frozen–thawed embryo, noninvasive chromosomal screening, preimplantation genetic testing, spent embryo culture medium, trophectoderm biopsy

## Abstract

**Background:**

Preimplantation genetic testing for aneuploidy (PGT-A) can improve embryo selection in assisted reproductive technology; however, the trophectoderm (TE) biopsy required for testing is invasive and may compromise embryo integrity. While noninvasive chromosomal screening (NICS) using cell-free DNA from spent embryo culture medium (SECM) is a promising alternative, its research in frozen–thawed embryos remains limited. We systematically evaluated and compared the detection performance of SECM-based NICS in fresh and frozen–thawed embryos and explored its clinical application potential. Overall, 104 SECM samples from 27 couples who were undergoing intracytoplasmic sperm injection–PGT-A were analyzed (65 from the fresh group and 39 from the frozen–thawed group). TE biopsy results were used as the gold standard for assessing NICS efficacy. A paired-sample design was employed to conduct synchronous sequencing analyses of TE biopsy cells, fresh SECM, frozen–thawed SECM, and whole-embryo samples from 24 embryos.

**Results:**

The overall detection success rate of the NICS was 98.08%. When TE biopsy was used as the gold standard, the overall sensitivity, specificity, and clinical consistency of the NICS were 88.46%, 50%, and 79.41%, respectively. Stratified analysis showed that, for SECM-NICS, frozen–thawed embryos demonstrated higher DNA library concentrations (*P* = 0.046), sensitivity (97.30% vs 78.05%, *P* = 0.008), positive predictive value (PPV; 100% vs 72.73%, *P* < 0.01), and clinical consistency (97.37% vs 68.75%, *P* = 0.001), with a lower rate of complete chromosomal inconsistency (2.63% vs 35.94%, *P* < 0.01), compared with fresh embryos. The high-concentration group (≥10 ng/µL) exhibited superior PPV and clinical consistency. Paired-sample analysis confirmed a high consistency between the TE biopsy and whole-embryo analysis results (clinical consistency: 100%). When whole-embryo sequencing was used as the absolute gold standard, the frozen–thawed SECM group showed a significantly lower rate of complete chromosomal inconsistency than the fresh group (4.35% vs 33.33%, *P* = 0.023). Mosaics were a key contributor to diagnostic discordance.

**Conclusion:**

The detection performance of NICS based on frozen–thawed SECM demonstrated superior sensitivity, PPV, and consistency with the actual chromosomal status compared with that using fresh embryos; this approach, as a noninvasive method, demonstrates potential for genetic assessment of cryopreserved embryos, potentially avoiding the need for secondary, invasive biopsies.

## Introduction

Preimplantation genetic testing for aneuploidy (PGT-A) is an essential method for embryo screening in assisted reproductive technology (ART). The conventional gold standard involves biopsy of the trophectoderm (TE) of the blastocysts (1, 2). However, TE biopsy has several limitations. First, the biopsy process may damage embryo developmental potential, and its technical complexity introduces risks of human error that may compromise embryo viability (3, 4). Second, TE cells may not accurately represent the chromosomal status of the entire embryo, as inconsistencies between TE and inner cell mass (ICM) or whole embryo have been reported (5–8). While TE biopsy is generally considered safe in the long term, with most large-scale studies reporting no significant increase in adverse neonatal outcomes, recent evidence suggests potential associations with obstetric complications, such as gestational hypertension and umbilical cord abnormalities (9, 10). Animal studies have also provided mechanistic insights into potential epigenetic modifications (11–14). Of note, embryologist training standards vary across regions, which may influence biopsy outcomes.

Recent studies have found that spent embryo culture medium (SECM) contains cell-free DNA (cfDNA) released by embryos, which can serve as a genetic material source for noninvasive chromosome screening (NICS). This approach offers a promising direction to overcome the limitations of traditional biopsy (15–19). Current NICS research is primarily focused on SECM from fresh embryos. Although the amplification success rate of cfDNA is generally high, reported consistency between NICS and TE biopsy varies widely, ranging from 33% to 100% (20).

In clinical practice, many patients have only frozen embryos available for PGT-A testing. Performing PGT-A on frozen embryos can reduce time and economic costs while avoiding repeated ovarian stimulation. However, invasive biopsy on frozen–thawed embryos may exacerbate cryodamage. Thus, developing NICS techniques for frozen–thawed embryos holds clinical significance. Current research on SECM-based NICS in frozen–thawed embryos remains limited. Huang et al. and Jiao et al. reported amplification success rates of 92.3% and 100% for frozen–thawed embryos, with clinical consistency rates of 93.8% and 90.48%, respectively (21, 22). Similarly, Li et al. achieved an amplification success rate of 97.4% and consistency of 87.2% (18). More recently, Ardestani et al. reported concordance rates between NICS and whole-embryo analysis ranging from 90.5% to 93.6% (23). Although these findings suggest higher success and concordance for frozen–thawed embryos than for fresh embryos, direct comparative data are lacking. A key question is whether the thawing process affects DNA quality and quantity in SECM, thereby influencing NICS accuracy.

In this study, we aimed to systematically evaluate and compare the detection performance of SECM-NICS in fresh and frozen–thawed embryos. Our NICS protocol included an additional step of blastocoele collapse prior to SECM collection to release blastocoel fluid-derived cfDNA; therefore, the procedure is technically minimally invasive. Further, we introduced two main innovations. First, we assessed the overall efficacy of SECM-NICS and examined the effects of embryo type, embryo quality, and DNA library concentration on detection outcomes. Second, we employed a paired-sample design to collect TE biopsy, fresh SECM, frozen–thawed SECM, and whole-embryo samples from the same embryo, enabling precise comparison while controlling for individual heterogeneity.

## Methods

### Research design and ethics

This study was approved by the Medical Ethics Committee of Zhongshan Boai Hospital (Approval No. KY-2021-012-43) and conducted in accordance with the Declaration of Helsinki. From January 2022 to December 2024, 27 couples who were undergoing intracytoplasmic sperm injection-PGT-A (ICSI-PGT-A) cycles were enrolled. A total of 71 blastocysts were cultured, and each underwent TE biopsy during the fresh cycle. SECM samples were collected and divided into fresh (n=65) and frozen–thawed (n=39) groups. TE biopsy PGT-A results served as the gold standard. Stratified analyses were conducted based on embryo type, quality, and DNA library concentration.

**Figure 1:**
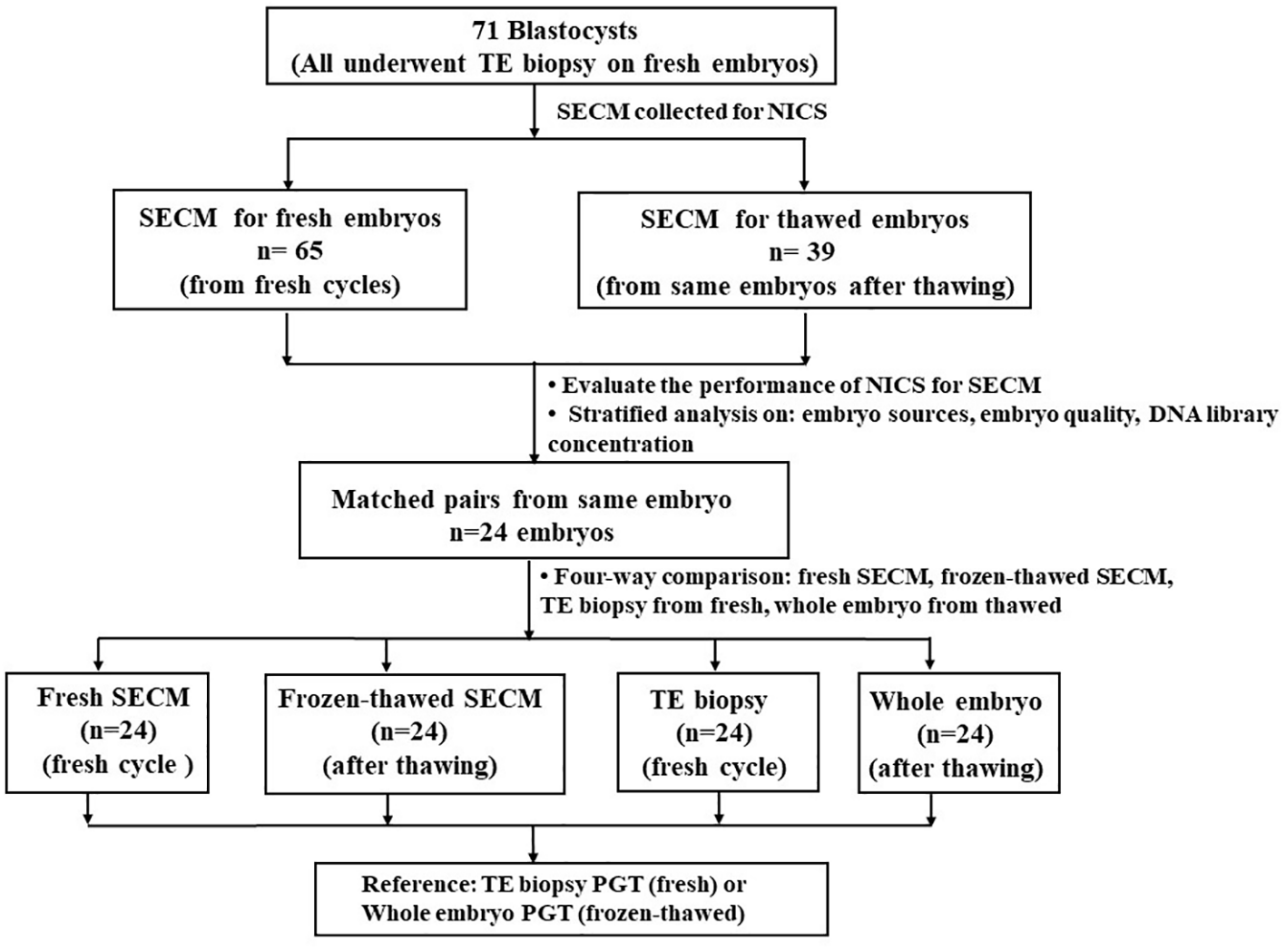
Workflow chart. PGT, preimplantation genetic testing; NICS, noninvasive chromosomal screening; TE, trophectoderm; SECM, spent embryo culture medium.

To control for inter-individual variation, a matched sample set of 24 embryos was established. For these samples, TE biopsy was performed on fresh embryos, and whole-embryo samples were collected after thawing. Thus, in the four-way comparison, fresh SECM and TE biopsy were derived from fresh cycles, while frozen–thawed SECM and whole embryos were derived from the same embryos after thawing (**Figure 1**).

### Embryo culture, TE biopsy, collection of fresh SECM, and embryo freezing

Oocytes were treated with 40 IU hyaluronidase for 2–3 min to remove cumulus cells, followed by ICSI. Fertilization was assessed 18–20 h post-ICSI. On day 3, residual cumulus cells were removed. Embryos were then cultured in G-2 Medium (Vitrolife) at 37 °C, 5% CO₂, 5% O₂. On day 4, the culture medium was refreshed. Embryo quality was assessed on days 5 or 6 using the Gardner scoring system (good-quality: AA or AB/BA; moderate/poor: BB/AC/CA/BC/CB). TE biopsy was performed using laser assistance (24). Biopsied embryos were vitrified within one hour. Fresh SECM was collected by aspirating culture droplets three times and transferring to pre-labeled PCR tubes with PBS buffer, then stored at –20 °C.

### Embryo thawing, collection of thawing embryo culture medium, and whole-embryo collection

Frozen embryos were thawed using a commercial kit (Vitrolife). After thawing, embryos were cultured in G-2 Medium for 6 h. Laser-assisted shrinkage was then performed to release blastocoel fluid. This blastocoele collapse step involves additional embryo manipulation beyond simply collecting culture medium, making this approach minimally invasive. The culture medium containing this fluid was collected into PCR tubes. Shrunken embryos were transferred to separate PCR tubes for whole-embryo analysis. All samples were collected with patient-informed consent.

### Whole-genome amplification, quantification, next-generation sequencing, and data analysis

All samples (SECM, TE biopsy cells, whole embryos) were amplified using MALBAC (Yikon Gene). Amplified products were purified, end-repaired, A-tailed, adapter-ligated, and library-enriched by PCR. Library concentrations were quantified using the Qubit dsDNA HS kit (Thermo Fisher Scientific) on a Qubit 4.0 fluorometer.

Sequencing was performed on an Illumina Dx platform (PE150). After quality control with Fastp, reads were aligned to hg19 using BWA, PCR duplicates removed with Picard, and aneuploidy analyzed using ChromGo software (Yikon Gene).

### Outcome assessment

Detection success rate: proportion of samples yielding valid NGS data. DNA library concentration was quantified as above: TP: SECM and reference both aneuploid; FP: SECM aneuploid, reference euploid; FN: SECM euploid, reference aneuploid; TN: both euploid. Sensitivity = true positive (TP)/[TP+false negative (FN)]; specificity = true negative (TN)/[TN+false positive (FP)]; PPV = TP/(TP+FP); negative predictive value (NPV) = TN/(TN+FN). Clinical consistency refers to agreement at the clinical diagnostic level (euploid vs. aneuploid/mosaic), not at the individual chromosome level. Complete chromosomal inconsistency: no shared abnormalities; partial consistency: at least one shared abnormality; complete consistency: full alignment across all chromosomes.

### Statistical analysis

Data analysis was conducted using SPSS statistical software (version 27.0). Continuous variables (such as DNA library concentrations) are expressed as means ± standard deviation, and inter-group comparisons were performed using *t-tests*. Categorical variables (such as detection success rates, sensitivity, specificity, PPV, NPV, clinical consistency, complete chromosomal inconsistency, partial chromosomal consistency, and complete chromosome consistency) are expressed as percentages, with intergroup comparisons performed using chi-squared tests or the Fisher exact tests. A *P* value of <0.05 was considered statistically significant.

## Results

### Performance evaluation of SECM-NICS using TE biopsy results as the reference standard

The performance of SECM detection was evaluated using TE biopsy results as the reference standard. Furthermore, a stratified analysis was conducted to assess the impact of embryo type, embryo quality, and DNA library concentration on SECM-NICS performance. Overall, 104 SECM samples were subjected to NICS detection, yielding a detection success rate of 98.08% (102/104) and a mean DNA library concentration of 16.01 ± 13.82 ng/μL (**Table 1**). Compared with TE biopsy results, SECM-NICS demonstrated a sensitivity, specificity, PPV, NPV, and clinical consistency of 88.46%, 50%, 85.19%, 57.14%, and 79.41%, respectively (i.e., agreement at the diagnostic outcome level, classifying samples as euploid vs. aneuploid/mosaic). A detailed comparison of chromosomal consistency between SECM-NICS and TE biopsy results revealed that the proportions of complete chromosomal inconsistency, partial consistency, and complete consistency were 23.53%, 44.12%, and 32.35%, respectively.

**Table 1:** Considering the TE biopsy results as the gold standard, the performance of SECM results and TE biopsy results was compared, and the SECM results were stratified and compared in terms of embryo type, embryo quality and DNA library concentration.

Item	The overall outcome of SECM	Embryo type			Embryo quality			DNA library concentration		
		SECM of fresh embryos	SECM of frozen–thawed embryos	P	SECM of good-quality embryos	SECM of moderate- or poor-quality embryos	P	SECM of DNA library concentration ≥10 ng/μl	SECM of DNA library concentration <10 ng/μl	P
Detection success rate (%)	98.08% (102/104)	98.46% (64/65)	97.46% (38/39)	1.000	97.10% (67/69)	100% (35/35)	0.920	100% (58/58)	95.65% (44/46)	0.874
DNA library concentration, (ng/μl)	16.01 ± 13.82	13.92 ± 14.86	19.49 ± 11.23	0.046	14.30 ± 13.51	19.37 ± 14.00	0.077	25.56 ± 11.27	3.97 ± 3.25	<0.01
Sensitivity (%)	88.46% (77.8–93.1)	78.05% (62.8–88.3)	97.30% (84.2–99.9)	0.008	85.11% (72.0–92.9)	90.32% (74.8–96.9)	0.732	88.37% (75.2–95.0)	83.33% (65.3–93.2)	1.000
Specificity (%)	50% (31.2–68.8)	47.83% (28.9–67.5)	100.00% (-)	0.458	50.00%(27.5–68.5)	50.00% (-)	1.000	62.50% (–)	40.00% (19.8–64.3)	0.400
PPV (%)	85.19% (75.5–91.4)	72.73% (57.9–83.9)	100.00% (90.3–100.0)	<0.01	80.00% (65.2–87.7)	93.33% (77.9–98.5)	0.194	92.68% (80.1–97.8)	73.53% (56.0–86.0)	0.024
NPV (%)	57.14% (34.5–73.3)	55.00% (34.2–74.2)	50.00% (-)	1.00	58.82% (33.7–75.5)	40.00% (-)	0.624	50.00% (23.70–76.30)	54.55% (28.0–78.8)	0.670
Clinical consistency (%)	79.41% (81/102)(70.5–86.3)	68.75% (44/64) (55.6–77.9)	97.37% (37/38)(81.8–98.9)	0.001	76.12% (51/67) (64.6–84.9)	85.71%(30/35)(69.5–94.3)	0.310	94.83% (55/58) (85.7–98.3)	72.73% (32/44)(58.1–83.7)	0.002
Complete chromosomal inconsistency (%)	23.53% (24/102)	35.94% (23/64)	2.63% (1/38)	<0.01	26.87% (18/67)	17.14% (6/35)	0.331	15.52% (9/58)	34.09% (15/44)	0.035
Partial chromosomal consistency (%)	44.12% (45/102)	40.63% (26/64)	50.00% (19/38)	0.412	37.31% (25/67)	57.14% (20/35)	0.062	44.83% (26/58)	43.18% (19/44)	1.000
Complete chromosome consistency (%)	32.35% (33/102)	23.44% (15/64)	47.37% (18/38)	0.412	35.82% (24/67)	25.71% (9/35)	0.375	39.66% (23/58)	22.73% (10/44)	0.089

*SECM, spent embryo culture media; TE, trophectoderm.

*CI, 95% confidence interval calculated using the Wilson Score method. Data in parentheses are 95% confidence intervals for sensitivity, specificity, positive predictive value (PPV), negative predictive value (NPV), and clinical consistency. “(-)” indicates that the confidence interval was not calculated due to limited sample size (denominator < 10).

*PPV of SECM of frozen–thawed embryos is reported without confidence intervals because the point estimate is 100% (zero variance).

*Good quality: AA/AB/BA; Moderate- or poor quality: BB/AC/CA/BC/CB;.

*Sensitivity, true positive (TP)/(TP + false negative (FN)); Specificity: true negative (TN)/(TN + false positive (FP)); PPV: TP/(TP + FP).

NPV, TN/(TN + FN).

*Clinical consistency: agreement between noninvasive chromosomal screening (NICS) and TE biopsy at the level of clinical diagnosis (euploid vs. aneuploid/mosaic), not at the individual chromosome level.

Stratified analysis by embryo type revealed that frozen–thawed embryos exhibited significantly higher DNA library concentrations (19.49 ± 11.23 ng/μL vs. 13.92 ± 14.86 ng/μL, *P* = 0.046), sensitivity (97.30% vs. 78.05%, *P* = 0.008), PPV (100% vs. 72.73%, *P* < 0.01), and clinical consistency (97.37% vs. 68.75%, *P* = 0.001) in SECM-NICS detection than did fresh embryos, with all differences being statistically significant. Furthermore, the proportion of complete chromosomal discordance was significantly lower in the frozen–thawed group than in the fresh embryo group (2.63% vs. 35.94%, *P* < 0.01). No significant differences were observed in the detection success rate (98.46% vs. 97.46%), specificity (47.83% vs. 100.00%), NPV (55.00% vs. 50.00%), partial chromosomal concordance (40.63% vs. 50.00%), and complete concordance (23.44% vs. 47.37%) between the two groups.

Among the 104 SECM samples, 69 (66.3%) were from good-quality blastocysts (AA or AB/BA) and 35 (33.7%) from moderate or poor-quality blastocysts (BB/AC, or CA, BC/CB). Stratified analysis by embryo quality showed no statistical differences between high- and medium/poor-quality embryos in the detection success rate (97.10% vs. 100%, P = 1.000), DNA library concentration (14.30 ± 13.51 ng/μL vs. 19.37 ± 14.00 ng/μL, P = 0.077), sensitivity (85.11% vs. 90.32%, P = 0.732), specificity (50.00% vs. 50.00%, P = 1.000), PPV (80.00% vs. 93.33%, P = 0.194), NPV (58.82% vs. 40.00%, P = 0.624), clinical consistency (76.12% vs. 85.71%, P = 0.310), and complete chromosomal inconsistency (26.87% vs. 17.14%, P = 0.331), partial chromosomal consistency (37.31% vs. 57.14%, P = 0.062), or complete chromosomal consistency (35.82% vs. 25.71%, P = 0.375). Although not statistically significant, the medium/poor-quality group showed a slightly upward trend in both the DNA library concentration and the proportion of partially consistent chromosomes. Stratified analysis by DNA library concentration indicated that the high-concentration group (≥10 ng/µL) significantly outperformed the low-concentration group (<10 ng/µL) in terms of PPV (92.68% vs. 73.53%, *P* = 0.024) and clinical consistency (94.83% vs. 72.73%, *P* = 0.002). Similarly, the high-concentration group demonstrated a significantly lower proportion of completely inconsistent chromosomes than the low-concentration group (15.52% vs. 34.09%, *P* = 0.035), with statistically significant differences.

### Paired-sample comparison and cross-validation of SECM-NICS with TE biopsy and whole-embryo analysis results

Using the TE biopsy and whole-embryo analyses results as reference standards, performance differences in SECM detection between fresh and frozen–thawed embryos were compared. To eliminate the potential impact of individual embryo differences on the results, a paired-sample analysis was performed using matched samples derived from the same embryo (including TE biopsy cells, fresh embryo SECM, frozen–thawed embryo SECM, and whole embryos) while concurrently validating the consistency between TE biopsy and whole-embryo analyses.

The SECM detection success rates for fresh and frozen–thawed embryos were similar (24 and 23 cases, respectively), with no significant difference in DNA library concentration (21.63 ± 16.39 ng/μL vs 17.95 ± 11.16 ng/μL, *P* = 0.368) (**Table 2**). A high degree of concordance was observed between TE biopsy and whole-embryo analysis results, with 100% clinical consistency, 0% complete chromosomal inconsistency, and 87.5% complete chromosomal consistency. Based on these results, regardless of whether TE biopsy or whole-embryo analysis was used as the reference standard, the frozen–thawed embryo group demonstrated superior sensitivity (95.65% vs 75.00%, *P* = 0.097) and clinical consistency (95.65% vs 75.00%, *P* = 0.097), compared with the fresh embryo group. However, the differences were not statistically significant. Both groups exhibited a PPV of 100% when TE biopsy was used as the reference standard. Chromosomal analysis revealed that the rate of complete chromosomal discordance was numerically higher in the fresh embryo group than in the frozen–thawed embryo group (25.00% vs 4.35%), although the difference was not significant (P = 0.097)—likely due to the limited sample size (n=24). Notably, when whole-embryo analysis was used as the reference standard, the rate of complete chromosomal discordance was significantly higher in the fresh embryo group than in the frozen–thawed embryo group (33.33% vs 4.35%, P = 0.023). The effect size for clinical consistency between the two groups was 20.65% (95.65% vs 75.00%), which is clinically reasonable despite not reaching statistical significance. No significant differences were observed between the two groups in terms of partial or complete concordance. **Table 3** presents the chromosomal karyotype results of the 24 blastocysts, including both fresh and frozen–thawed embryo SECM, TE biopsy cells, and whole embryos, further assessing the consistency among different samples.

**Table 2:** Considering the results of TE biopsy and whole embryo as the gold standards respectively, the performance results of SECM detection for fresh embryos and frozen–thawed embryos were compared.

Items	TE biopsy as the gold standards			Whole embryo as the gold standards			
	SECM of fresh embryos	SECM of frozen–thawed embryos	P	SECM of fresh embryos	SECM of frozen–thawed embryos	P	TE biopsy
Detection success rate (%)	100% (24/24)	95.83% (23/24)	–	100% (24/24)	95.83% (23/24)	–	24
DNA library concentration (ng/μl)	21.63 ± 16.39	17.95 ± 11.16	0.368	21.63 ± 16.39	17.95 ± 11.16	0.368	–
Sensitivity (%)	75.00% (55.1–88.0)	95.65% (76.0–99.8)	0.097	83.33% (62.6–94.3)	95.65% (76.0–99.8)	0.097	/
Specificity (%)	/	/		/	/		/
PPV (%)	100%	100%	–	100%	100%	–	/
NPV (%)	/	/		/	/		/
Clinical consistency (%)	75.00% (18/24) (55.1–88.0)	95.65% (22/23) (76.0–99.8)	0.097	75% (18/24) (55.1–88.0)	95.65% (22/23) (76.0–99.8)	0.097	100.00% (24/24)
Complete chromosomal inconsistency (%)	25.00% (6/24)	4.35% (1/23)	0.097	33.33% (8/24)	4.35% (1/23)	0.023	0
Partial chromosomal consistency (%)	62.5% (15/24)	56.52% (13/23)	0.770	45.83% (11/24)	56.52% (13/23)	0.564	12.50% (3/24)
Complete chromosome consistency (%)	12.5% (3/24)	39.13% (9/23)	0.770	20.83% (5/24)	39.13% (9/23)	0.212	87.5% (21/24)

*Data in parentheses are 95% confidence intervals (Wilson Score) for sensitivity and clinical consistency.

*Positive predictive value (PPV) is reported without confidence intervals because the point estimate is 100% (zero variance).

**Table 3:** Comparison of SECM of fresh embryos, SECM of frozen–thawed embryos, TE biopsy and chromosome analysis of whole embryos.

Blastocysts	Embryo quality	SECM of fresh embryos	SECM of frozen–thawed embryos	TE biopsy	Whole embryo	General consistency
1	4AB	46, XN, + 16 (×3, mos, ~50%)	46, XN, + 16, -22	46, XN, + 16 (×3), -22 (×1)	46, XN, + 16, -22	M/A/A/A
2	4AB	47, XY, + 4q (q24→q35.2, ~89Mb, ×3), -5q (×1, mos, ~70%), +16 (×3), +19q (×3)	47, XN, del (5) (q11.1q35.3) (~131.42Mb), +16, dup (19) (q11q13.43) (~31.13Mb, ~56%)	47, XN, -5q (q11.1→ q35.2, ~126Mb, ×1), +16 (×3), +19q (×3, mos, ~58%)	47, XN, del (5) (q11.1q35.3) (~131.52Mb), +16, dup (19) (q11q13.43) (~31.13Mb)	A/A/A/A
3	4BB	46, XN	46, XN, del (19) (p13.3p11) (~25.00Mb)	47, XXX, +X (×3), -1 (×1), -2, +3p (×4), +3q (×3), +4q (×3), +5p (p15.1→p14.1, ~7.9Mb, ×3), +6p (p25.3→p12.3, ~49Mb, ×3), -7q (q34→q35, ~4.1Mb, ×1), -8, -9p (pter→p21.3, ~25Mb, ×1), -10q (q25.1→q25.1, ~5.0Mb, ×1), +11 (×3), -13q (q32.3→q33.3, ~6.7Mb, ×1) , +15q (q21.1→q21.3, ~5.2Mb, ×3), +16q (q11.2→q12.1, ~4.3Mb, ×3), +17p (×3), -18p (×0), -18, -19 (×1), +22 (×3)	46, XN, dup (5) (p15.33p11) (~46.20Mb), del (19) (p13.3p11) (~24.60Mb)	E/A/A/A
4	4AA	46, XN, -6q (q21→q27, ~61Mb, ×1, mos, ~60%), +17q (×3, mos, ~60%)	47, XN, dup (7) (q31.1q36.3) (~45.14Mb), +17	47, XN, del (6) (q16.1q27) (~72.12Mb), +17	47, XN, + 17	M/A/A/A
5	4AA	46, XN, + 19 (×3, mos, ~60%)	46, XN, dup (19) (p13.3q13.42) (~55.50Mb, ~69%)	47, XN, + 19	47, XN, + 19	M/M/A/A
6	4AB	46, XN	47, XN, + 19, +22 (~51%)	47, XN, + 19	46, XN, dup (19) (p13.3p12) (~20.80Mb, ~68%), dup (19) (p12q13.43) (~35.13Mb)	E/A/A/A
7	4AB	46, XY, +Xq (q13.3→q21.33, ~24Mb, ×2), +1q (q25.2→q32.2, ~31Mb, ×3), -3q (q13.13→q29, ~88Mb, ×1, mos, ~50%), -11 (×1, mos, ~60%), -12q (q21.31→q21.33, ~11Mb, ×1), -15q (q21.3→q26.3, ~49Mb, ×1, mos, ~50%), +19p (×3, mos, ~50%), -21 (×1, mos, ~70%)	43, XN, dup (1) (q25.3q31.3) (~13.50Mb), -11, - 15, + 19 (~55%), -21	44, XN, -11, -15, +19, -21	44, XN, -11, -15, +19, -21	A/A/A/A
8	4BB	45, XN, -7 (×1), +9 (×3), +14q (q11.2→q22.1, ~34Mb, ×3), +14q (q23.3→q32.33, ~42Mb, ×3), -16 (×1)	46, XN, -3 (~68%), +6 (~61%), -7, +9, +14, -16	45, XN, -3, -7, +9, +14, -16	46, XN, -7, +9, +14, -16	A/A/A/A
9	4AB	46, XN, -3p (×1, mos, ~50%), -16 (×1, mos, ~50%)	46, XN, + 11 (~68%)	47, XN, + 11	47, XN, + 11	M/M/A/A
10	4AC	46, XN	46, XN, dup (5) (p15.33p13.3) (~29.50Mb)	46, XN, del (5) (p15.33p13.3) (~29.60Mb)	46, XN, del (5) (p15.33p13.3) (~29.60Mb)	E/A/A/A
11	4BB	46, XN, + 13q (q12.11→q21.2, ~42Mb, ×3, mos, ~60%)	46, XN, + 13 (~56%)	47, XN, + 13	47, XN, + 13	M/M/A/A
12	4BB	46, XN, -19q (×1, mos, ~50%)	45, XN, -19	45, XN, -19	45, XN, -19	M/A/A/A
13	5AA	47, XN, + 2 (×3)	47, XN, + 2	47, XN, + 2	47, XN, + 2	A/A/A/A
14	5AA	46, XN	48, XN, +X (~52%), +9, +11 (~60%), +16 (~56%), +18	48, XN, + 9, +18	48, XN, + 9, +18	A/A/A/A
15	4AA	48, XN, + 14 (×3), +16 (×3), +17p (×3, mos, ~70%), +17q (×3)	49, XN, -9 (~61%), +14, +16, +17	49, XN, + 14, +16, +17	49, XN, + 14, +16, +17	A/A/A/A
16	4AB	46, XN, + 16 (×3), -22 (×1)	46, XN, + 16, -22	46, XN, + 16, -22	46, XN, + 16, -22	A/A/A/A
17	3AB	46, XX, -Xp (p22.33→p11.4, ~37Mb, ×1), -Xq (q13.2→q28, ~82Mb, ×1), -6q (q22.33→q24.3, ~21Mb, ×1), +8q (q11.1→q12.1, ~10Mb, ×3), -13q (q14.11→q31.1, ~41Mb, ×1, mos, ~50%), +14q (q11.1→q21.1, ~23Mb, ×3)	NA	47, XN, + 8	47, XN, + 8	A/-/A/A
18	3BB	46, XY, - 7 (×1, mos, ~50%), +18p (×3)	48, XN, dup (1) (p36.33p11.2) (~120.50Mb, ~56%), - 2 (~47%), dup (6) (p24.3p22.3) (~10.50Mb), du p (6) (p21.1p11.2) (~17.00Mb), dup (6) (q12q22.31) (~58.00Mb), -7, +9, -10 (~54%), - 11 (~63%), dup (12) (p13.2p11.1) (~22.00Mb), dup (13) (q12.11q34) (~91.50Mb), +14, +18, - 20, + 22	47, XN, -7 (~56%), +18	47, XN, -7 (~66%), +18	A/A/A/A
19	4BB	46, XN, -7p (×1, mos, ~60%), - 7q (×1, mos, ~50%)	45, XN, -7	45, XN, -7	45, XN, -7	M/A/A/A
20	4AB	48, XN, -X (~55%), +2 (~63%), +4, -7, +11, +12, +13 (~61%), -15, +20, -22 (~65%)	46, XN	46, XN, dup (7) (q21.11q33) (~60.60Mb), dup (7) (q33q35) (~6.00Mb), del (7) (q35q36.3) (~15.34Mb)	46, XN, dup (7) (q21.11q33) (~60.60Mb), dup (7) (q33q35) (~6.00Mb), del (7) (q35q36.3) (~15.34Mb)	A/E/A/A
21	4AB	47, XN, del (9) (q21.11q34.3) (~70.71Mb, ~63%), +17	47, XN, + 17	47, XN, + 17	47, XN, + 17	A/A/A/A
22	4BC	46, XN, del (3) (p26.3p12.1) (~84.00Mb), +21 (~69%)	45, XN, del (3) (p26.3p12.1) (~84.00Mb, ~65%), -21	47, XN, del (21) (q21.1q22.3) (~31.13Mb)	45, XN, -21	A/A/A/A
23	4AA	46, XN,	46, XN, + 4, -18	46, XN, + 4, -18	46, XN, + 4, -18	E/A/A/A
24	4AB	46, XN,	49, XN, +X, +1, +2 (~56%), +5 (~68%), +12 (~62%), +13, +14 (~67%), +18 (~62%)	48, XN, + 5 (~52%), +13, +14, +18 (~53%)	46, XN, +X (~61%), +1 (~66%), +2 (~61%), +5 (~56%), +12 (~59%), +13 (~64%), +14 (~56%), +18 (~56%)	E/A/A/M

*General consistency (SECM of fresh embryos/SECM of frozen–thawed embryos/TE biopsy/whole embryo): “NA” represents not available, “A” represents Aneuploid, “E” represents Euploid, “M” represents Mosaic, “-” represents NA.

## Discussion

This study systematically evaluated the overall detection performance of NICS based on SECM using TE biopsy results as the reference standard. Furthermore, stratified analyses were performed to assess the effects of embryo type, embryo quality, and DNA library concentration. Additionally, for the first time, we employed a paired-sample design to compare, within the same embryo, the results of fresh SECM, frozen–thawed SECM, TE biopsy, and whole-embryo analysis. Notably, in our cohort, TE biopsy and whole-embryo analysis showed high consistency, with 100% clinical consistency, 0% complete chromosomal inconsistency, and 87.5% complete chromosomal consistency (**Table 2**). This finding suggests that, within this specific study population, TE biopsy served as a reasonably reliable reference standard, partially mitigating concerns about its use as a gold standard. The overall detection success rate of NICS in this study was extremely high (98.08%)—consistent with the high amplification success rates reported in several studies (21, 25–28). However, a high detection success rate does not necessarily equate to high diagnostic accuracy. Although the overall sensitivity of NICS in this study was acceptable (88.46%), the specificity was relatively low (50%), indicating that the current NICS technology is more effective at identifying aneuploidy than confirming euploidy. This phenomenon of low specificity has been reported in several previous studies of NICSs. For example, Handayani et al. and Sialakouma et al. collected 28 and 33 SECM cfDNA samples, and reported sensitivities of 100% and 91.6% and specificities of 13.3% and 76.19%, respectively (29, 30). The reduced specificity may be attributed to the complex sources of cfDNA in the culture medium, such as apoptotic cells and selectively released DNA fragments, leading to a relatively high risk of false positives (28). The overall clinical consistency between NICS and TE biopsy in this study was 79.41%—comparable to the results of an extensive multicenter study by Rubio et al., which involved more than 1, 000 biopsy blastocysts and their corresponding SECM cfDNA, with a mean clinical consistency of 78.2% (31). However, the complete concordance rate at the chromosomal level was only 32.35%. Of note, the reported consistency between NICS and TE biopsy varies widely across published studies, ranging from 33% to 100% (18, 20, 22, 32). Our finding of 79.41% clinical consistency falls within the upper-mid range of this spectrum—notably higher than the lower bound of 33% reported by Vera-Rodriguez et al. (32). This wide variability likely reflects differences in several key aspects of study designs, including: (1) cfDNA collection protocols (e.g., volume of culture medium, timing of collection, inclusion of blastocoele collapse); (2) amplification methods (e.g., MALBAC vs. other whole-genome amplification techniques); (3) sequencing platforms; (4) the criteria used to define “consistency” (e.g., clinical-level vs. chromosome-level agreement); and (5) embryo source (fresh vs. frozen–thawed embryos), which was identified by our stratified analysis as a major factor influencing NICS performance. Importantly, our study employed a paired-sample design and validated NICS results against whole-embryo sequencing as an absolute reference standard—methodological advances not present in most previous studies. These advances may partially explain the relatively high consistency observed in our cohort and provide a more rigorous benchmark for future NICS research.

The notably low specificity (50%) observed in this study warrants careful consideration. This finding indicates that while NICS is reasonably sensitive for detecting aneuploidy (88.46%), it has a substantial false-positive rate (50%) for classifying embryos as aneuploid when they are actually euploid. This limitation has direct clinical implications: FP results could lead to unnecessary discarding of viable embryos. Importantly, low specificity is not unique to our study. A recent large prospective study reported a specificity of only 50.7% for niPGT-A—consistent with our findings (33). Similarly, another study reported a specificity of 60% for aneuploidy detection using NiPGT-A (34). Handayani et al. also reported that the diagnostic potential of SECM cfDNA was only 50%, compared with TE biopsy, highlighting the challenge of low diagnostic accuracy (29). These findings suggest that low specificity is a common challenge across current NICS technologies rather than a problem specific to our cohort. Several factors may contribute to this low specificity (i.e., high FP rate). First, cfDNA in SECM originates from multiple cell types, including apoptotic inner cell mass cells whose chromosomal status may differ from that of the trophectoderm, creating biological noise that can lead to FP results. Second, technical noise inherent to whole-genome amplification of low-input DNA (mean 3.97 ng/μL in the low-concentration group) can introduce amplification bias and errors, leading to FP aneuploidy calls. Third, mosaicism in several embryos (e.g., blastocysts 1, 4, 5, 9, 11, 12, and 19) contributes to discordance between NICS and TE biopsy, in which NICS may detect mosaic signals not captured by TE biopsy, resulting in false positives (**Table 3**). Importantly, our stratified analysis identified DNA library concentration as a modifiable factor affecting NICS accuracy. The high-concentration group (≥10 ng/μL) demonstrated significantly better PPV (92.68% vs 73.53%, *P* = 0.024) and clinical consistency (94.83% vs 72.73%, *P* = 0.002) with a lower rate of complete chromosomal inconsistency (15.52% vs 34.09%, *P* = 0.035). This suggests that establishing a quality control threshold (e.g., ≥10 ng/μL) could reduce FP rates. Based on the current data, NICS should not replace TE biopsy for clinical decision-making. Potential applications of NICS are currently limited to: (a) as a preliminary screening tool; (b) for patients with only cryopreserved embryos who decline repeat biopsy, as an alternative after full disclosure of limitations; or (c) as a salvage test following TE biopsy failure.

The observed difference in consistency between NICS and TE biopsy results may primarily stem from fundamental differences in the targets of detection. TE biopsy primarily reflects the genomic information of local trophoblast cells, whereas the cfDNA in SECM originates from a mixture of DNA released by various embryonic cells, including apoptotic trophoblast cells, apoptotic ICM cells, cellular debris, or degraded DNA fragments. Furthermore, the low rate of complete chromosomal concordance is highly correlated with the presence of mosaicism, which further exacerbates the differences between TE biopsy and SECM results (20).

Embryo type is a significant factor that contributes to differences in NICS performance. Stratified analysis showed that frozen–thawed embryos exhibited significantly superior results across all key performance indicators, compared with fresh embryos, including a higher DNA library concentration (*P* = 0.046), sensitivity (97.30% vs 78.05%, p=0.008), PPV (100% vs 72.73%, p<0.01), and clinical consistency (97.37% vs 68.75%, p=0.001), along with a substantially lower rate of complete chromosomal inconsistency (2.63% vs 35.94%, *P* < 0.01). This result strongly suggests that the freeze–thaw process may enhance the release of more representative DNA into the culture medium by altering cell membrane permeability or increasing apoptosis, thereby improving the quality and quantity of cfDNA (35, 36). This is consistent with the findings of Huang et al. and Jiao et al., who reported higher amplification success rates and clinical concordance in NICS of frozen–thawed embryos (21, 22). Collectively, these results indicate that NICS may represent a promising alternative for the genetic assessment of cryopreserved embryos. It should be noted, however, that the freeze–thaw process itself may induce cellular damage and release of cfDNA, potentially creating a technical artifact that could confound direct comparisons with fresh embryos. The observed superior performance of frozen–thawed SECM may therefore reflect both biological factors (e.g., altered cell membrane permeability and increased apoptosis) and technical factors (e.g., increased cfDNA yield from damaged cells). The direction of this bias likely favors the frozen–thawed group, which may explain the higher DNA library concentrations, sensitivity, PPV, and clinical consistency observed in this group. Nevertheless, from a clinical perspective, the finding that frozen–thawed SECM yields higher-quality cfDNA for NICS analysis is practically useful regardless of the underlying mechanism.

In this study, embryo quality (good vs moderate or poor) did not significantly affect the performance of NICS, including the detection success rate, DNA concentration, sensitivity, specificity, or consistency, with no statistical differences between the two groups. This indicates that once embryos reach the blastocyst stage and are suitable for biopsy and SECM collection, embryo quality is not a critical factor in determining the success or accuracy of NICS. This is consistent with the findings of Zhang et al., who reported no correlation between embryo quality and cfDNA quantity (37). Therefore, for embryos of poor quality, it may be advisable to consider SECM for detecting chromosomal aneuploidy to minimize the damage caused by sampling procedures. Notably, the DNA library concentration was confirmed to be a significant technical parameter affecting NICS accuracy. We found that the high-concentration group demonstrated significantly superior PPV and clinical consistency, as well as a lower rate of complete chromosomal inconsistency. This suggests that obtaining a sufficient amount of cfDNA is a prerequisite for ensuring the reliability of NICS results. Several mechanisms may explain this observation. First, higher cfDNA input reduces amplification bias and allele dropout during whole-genome amplification, leading to more uniform genome coverage and fewer FP or FN calls. Second, higher DNA input provides a better signal-to-noise ratio, making true aneuploidy detection more reliable and reducing background noise. Third, higher cfDNA yield may indirectly reflect better embryo metabolic activity or cellular integrity, though this requires further investigation. Therefore, in future clinical applications, the DNA library concentration should be established as a key quality control parameter for ensuring the accuracy of NICS. Based on our stratified analysis, we propose that a threshold of ≥10 ng/μL can be used as a reference: samples with DNA library concentrations above this threshold demonstrated significantly better PPV (92.68% vs. 73.53%) and clinical consistency (94.83% vs. 72.73%), while samples below this threshold showed significantly lower reliability and should be interpreted with caution.

In this study, a unique paired sample design was employed to conduct a synchronous analysis of TE biopsies, fresh-embryo SECM, frozen–thawed SECM, and whole embryos derived from the same embryo. When evaluated against TE biopsy as the reference standard, frozen–thawed embryo NICS demonstrated a clear performance advantage, with higher sensitivity (95.65% vs 75.00%) and clinical consistency (95.65% vs 75.00%), compared with the fresh embryo group. Although the limited paired-sample size prevented statistical significance (*P* = 0.097), this trend was consistent with the results of the larger cohort analysis (**Table 1**), reinforcing the hypothesis that the freeze–thaw process enhances the ability to detect aneuploidy in cfDNA in SECM. Notably, when using the whole embryo as the absolute reference standard, frozen–thawed NICS exhibited a significantly lower rate of complete chromosomal inconsistency than fresh NICS (4.35% vs 33.33%, *P* = 0.023). This result confirms that frozen–thawed NICS has a higher intrinsic accuracy in reflecting the overall chromosomal status of the embryo. Furthermore, this study confirmed high consistency between TE biopsy and whole-embryo analysis (clinical consistency: 100%; complete chromosome consistency: 87.5%). This finding supports the reliability of TE biopsy as the reference standard in this cohort. It suggests that the discrepancies between NICS and TE biopsy are primarily attributable to the characteristics of cfDNA in SECM and technical limitations, rather than the insufficient representativeness of TE biopsy.

Mosaicism likely represents a major factor contributing to inconsistent diagnostic results (**Table 3**). Several embryos (e.g., blastocysts 1, 4, 5, 9, 11, 12, and 19) exhibited mosaic signals in at least one sample (marked as “M”). Based on the data in **Table 3**, we identified two typical patterns. First, NICS detected mosaic signals while TE biopsy and whole-embryo analysis reported uniform euploidy/aneuploidy (e.g., blastocysts 1, 4, 5, 9, 11, 12, and 19). As whole-embryo analysis confirmed the true chromosomal status of these embryos as uniform aneuploidy or euploidy, the mosaic signals detected by NICS likely represent FP artifacts arising from technical factors rather than genuine biological mosaicism. Potential technical causes include: (a) amplification bias during whole-genome amplification of low-input cfDNA, leading to uneven coverage across chromosomal regions; (b) maternal DNA contamination or background noise interfering with signal ratios. Second, TE biopsy detected mosaic signals, whereas NICS did not (e.g., blastocyst 24), which may occur when cfDNA concentration is too low or the proportion of mosaic cells falls below the detection threshold. These mechanisms highlight the inherent challenges in interpreting NICS results in the context of mosaicism.

This study has some limitations. First, the paired-sample analysis included a relatively small cohort (n=24), resulting in insufficient statistical power for some comparisons (e.g., the difference in sensitivity between fresh and frozen–thawed groups in the paired analysis did not reach statistical significance, P = 0.097). Nevertheless, our paired design—analyzing fresh SECM, frozen–thawed SECM, TE biopsy, and whole embryos from the same embryo—controls for inter-embryo variability, providing greater statistical efficiency for internal comparisons than unpaired designs with larger sample sizes. Second, all paired discarded embryos were confirmed to be aneuploid or mosaic through whole-embryo analysis; no euploid (negative) samples were included. This sample composition allowed effective evaluation of sensitivity and PPV but precluded calculation of specificity and NPV for euploid detection. Third, the low specificity (50%) in the overall analysis limits the clinical applicability of NICS as a standalone diagnostic test. Fourth, the potential for technical artifacts due to the freeze–thaw process (e.g., increased cfDNA release from damaged cells) cannot be fully excluded as a confounding factor. Fifth, without single-cell analysis, definitive classification of mosaicism versus technical noise remains challenging. Sixth, strictly speaking, our NICS protocol included blastocoele collapse to increase cfDNA yield. This additional embryo manipulation means the procedure should be classified as minimally invasive rather than completely noninvasive. Therefore, the conclusions of this study are primarily exploratory and hypothesis-generating, requiring validation in larger, prospective cohorts that include balanced numbers of euploid samples.

## Conclusions

Through a systematic comparative analysis, this study demonstrated that NICS based on frozen–thawed SECM exhibits superior detection performance compared with that using fresh SECM, including higher sensitivity, PPV, and concordance with whole-embryo karyotypes. For patients possessing only cryopreserved embryos and requiring genetic assessment, NICS shows promise as a noninvasive alternative that could potentially avoid secondary invasive biopsy. However, the limited sample size for paired analyses (n=24) and the low specificity (50%) preclude definitive conclusions about clinical readiness. Future large-scale, prospective studies with euploid controls are needed to validate the specificity of NICS, to establish robust quality control thresholds (e.g., DNA library concentration ≥10 ng/μL), and to further elucidate the molecular mechanisms by which the freeze–thaw process affects cfDNA quality before clinical implementation can be recommended.

## Data Availability

The data that support the findings of this study have been deposited into CNGB Sequence Archive (CNSA) of China NationalGeneBank DataBase (CNGBdb) with accession number CNP0009622.
